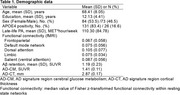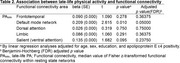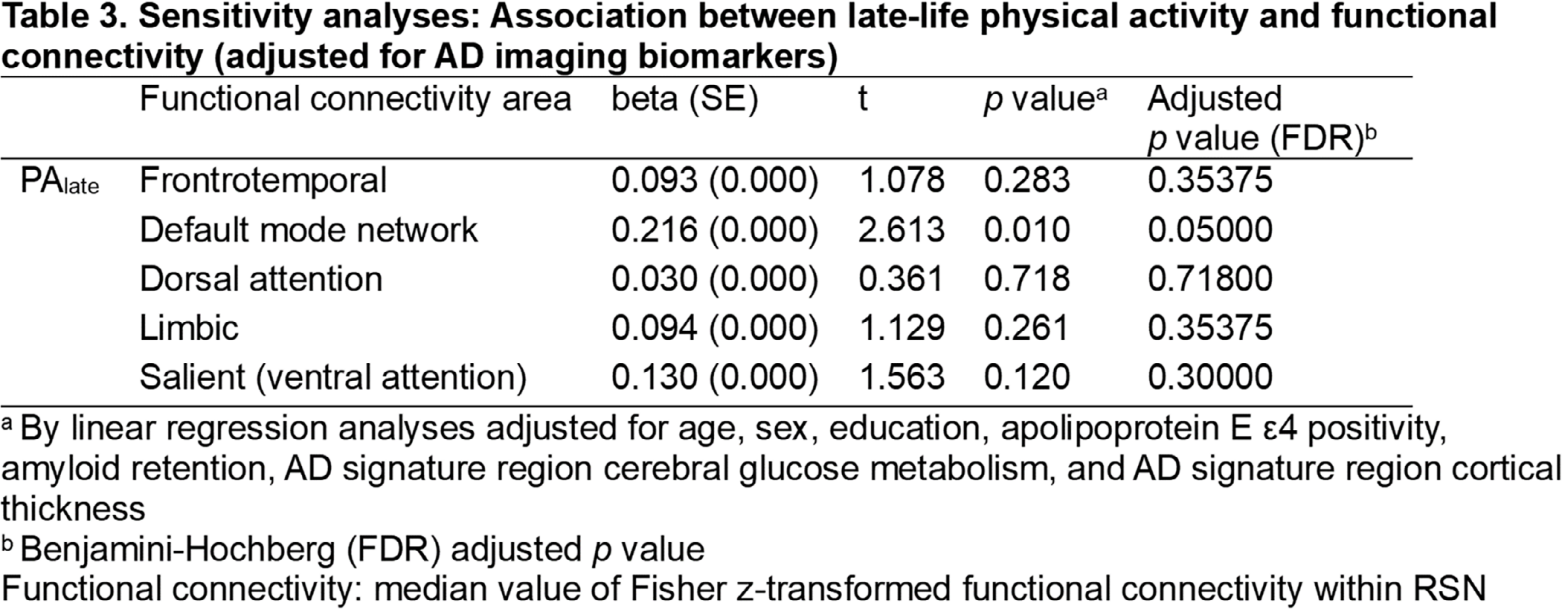# Late‐life physical activity and increased default mode network connectivity

**DOI:** 10.1002/alz70856_106382

**Published:** 2026-01-09

**Authors:** Bo Kyung Sohn, Min Soo Byun, Dahyun Yi, So Yeon Jeon, Yoon Young Chang, Hyeji Choi, Gijung Jung, Hyejin Ahn, Woo‐Jin Cha, Dong Woo Lee, Jun‐Young Lee, Yu Kyeong Kim, Koung Mi Kang, Chul‐Ho Sohn, Shannon L Risacher, Evgeny J. Chumin, Olaf Sporns, Kwangsik Nho, Andrew J. Saykin, Dong Young Lee, The KBASE Research Group

**Affiliations:** ^1^ Inje University Sanggye Paik Hospital, Seoul, Korea, Republic of (South); ^2^ Department of Neuropsychiatry, Seoul National University Hospital, Seoul, Korea, Republic of (South); ^3^ Department of Psychiatry, Seoul National University College of Medicine, Seoul, Korea, Republic of (South); ^4^ Seoul National University Medical Research Center, Seoul, Korea, Republic of (South); ^5^ Chungnam National University Hospital, Daejeon, Daejeon, Korea, Republic of (South); ^6^ Inje University Sanggye Paik Hosiptal, Seoul, Korea, Republic of (South); ^7^ Seoul National University Hospital, Seoul, Korea, Republic of (South); ^8^ Institute of Human Behavioral Medicine, Medical Research Center, Seoul National University, Seoul, Korea, Republic of (South); ^9^ Interdisciplinary program of cognitive science, Seoul National University College of Humanities, Seoul, Korea, Republic of (South); ^10^ Department of Neuropsychiatry, SMG‐SNU Boramae Medical Center, Seoul, Korea, Republic of (South); ^11^ Seoul National University College of Medicine, Seoul, Korea, Republic of (South); ^12^ SMG‐SNU Boramae Medical Center, Seoul, Korea, Republic of (South); ^13^ Department of Nuclear Medicine, Seoul National University College of Medicine, Seoul, Korea, Republic of (South); ^14^ Department of Radiology, Seoul National University Hospital, Seoul, Korea, Republic of (South); ^15^ Department of Radiology and Imaging Sciences, Indiana University School of Medicine, Indianapolis, IN, USA; ^16^ Indiana Alzheimer's Disease Research Center, Indiana University School of Medicine, Indianapolis, IN, USA; ^17^ Indiana Alzheimer's Disease Research Center, Indiana University School of Medicine, Indianapolis, IN, USA; ^18^ Center for Neuroimaging, Department of Radiology and Imaging Sciences, Indiana University School of Medicine, Indianapolis, IN, USA; ^19^ Department of Psychological and Brain Sciences, Indiana University, Bloomington, IN, USA; ^20^ Indiana Alzheimer's Disease Research Center, Indianapolis, IN, USA; ^21^ Center for Computational Biology and Bioinformatics, Indiana University School of Medicine, Indianapolis, IN, USA; ^22^ Center for Neuroimaging, Department of Radiology and Imaging Sciences, Indiana University School of Medicine, Indianapolis, IN, USA; ^23^ Department of Medical and Molecular Genetics, Indiana University School of Medicine, Indianapolis, IN, USA; ^24^ Center for Neuroimaging, Indiana University School of Medicine, Indianapolis, IN, USA

## Abstract

**Background:**

Higher levels of physical activity (PA) have been linked to a reduced risk of Alzheimer's disease (AD) dementia among older adults. However, the mechanisms underlying the association between increased PA and a lower risk of AD‐related cognitive decline remain unclear. This study aimed to investigate the relationship between late‐life PA (PA_late_) and functional connectivity (FC) within major functional resting state brain networks in cognitively healthy older adults.

**Method:**

This study was part of the Korean Brain Aging Study for Early Diagnosis and Prediction of Alzheimer's Disease (KBASE). It included 157 cognitively normal (CN) participants who underwent comprehensive clinical assessments at baseline. The participants also received multimodal brain imaging including resting‐state functional MRI, structural MRI, [^11^C] Pittsburgh Compound B (PiB) positron emission tomography (PET), [^18^F] fluorodeoxyglucose (FDG) PET, as well as an evaluation for PA_late_. For major functional resting state networks (RSN) including the frontotemporal, default mode, dorsal attention, limbic, and salience (ventral attention) networks, within‐system FC was calculated as the median value of Fisher z‐transformed node‐to‐node functional connectivity within each network.

**Result:**

PA_late_ demonstrated a significant positive association with FC of the default mode network (beta = 0.216, *p* = 0.010). No significant associations were found in other networks. The association between PA_late_ and the default mode network FC remained significant even after controlling for AD imaging biomarkers, including global amyloid retention, AD signature region cerebral glucose metabolism, and AD signature region cortical thickness.

**Conclusion:**

These findings indicate that PA_late_ is associated with increased functional connectivity within the default mode network, independent of AD‐related brain pathologies. While further research is needed, these findings suggest a potential role in reducing the risk of AD and related cognitive decline.